# Predictors of Changes in Sleep Duration in Dutch Primary Schoolchildren: the ChecKid Study

**DOI:** 10.1007/s12529-020-09876-7

**Published:** 2020-04-21

**Authors:** Nina L. Komrij, Maartje M. van Stralen, Vincent Busch, Maj-Britt M. R. Inhulsen, Maaike Koning, Elske de Jong, Carry M. Renders

**Affiliations:** 1 Department of Health Sciences, Faculty of Science, Amsterdam Public Health Research Institute, Vrije Universiteit, Amsterdam, The Netherlands; 2 Department of Epidemiology & Health Promotion, Youth Section, Municipal Health Service Amsterdam, Amsterdam, The Netherlands; 3 Research Centre Healthy Cities, Knowledge Centre for Health and Social Work, Windesheim University of Applied Sciences, Zwolle, The Netherlands

**Keywords:** Sleep, Child health, Determinants, Socioeconomic factors, Healthy lifestyle, Home environmental factors

## Abstract

**Background:**

Healthy sleep duration is essential to health and well-being in childhood and later life. Unfortunately, recent evidence shows a decline in sleep duration among children. Although effective interventions promoting healthy sleep duration require insight into its predictors, data on these factors are scarce. This study therefore investigated (i) which individual (lifestyle), social and cultural factors, and living conditions and (ii) which changes in these factors might be associated with the changes in sleep duration of Dutch primary schoolchildren observed over time.

**Method:**

Data from the ChecKid study was used, a dynamic cohort study among 4–13-year-old children living in the city of Zwolle, the Netherlands. Associations between changes in sleep duration and individual (lifestyle) factors (i.e., age, sex, physical activity behavior, sugar-sweetened beverage consumption, screen behavior), social and cultural factors (i.e., parental rules, ethnicity), and living conditions (i.e., parental education, presence of screens in the bedroom, household size) were analyzed using multivariable linear regression.

**Results:**

A total of 1180 children participated, aged 6.6 ± 1.4 years in 2009. Mean sleep duration decreased from 11.4 ± 0.5 h/night in 2009 to 11.0 ± 0.5 h/night in 2012. Older children, boys, children who used screens after dinner, children with greater computer/game console use, and children whose parents had low levels of education had a greater decrease in sleep duration.

**Conclusions:**

This article reports on one of the first large, longitudinal cohort studies on predictors of child sleep duration. The results of the study can inform future interventions aimed at promoting healthy sleep in primary schoolchildren.

## Introduction

Healthy sleep behavior, including adequate sleep duration, is essential to the development, well-being, and health of children [1, 2]. Several studies have demonstrated that inadequate sleep duration in children is associated with a wide spectrum of problems in childhood and later life, including obesity, diabetes, depression, and other mental health issues. Sleep duration is vital to brain and cognitive development, as well as to learning abilities, memory processes, school performance, and healthy physical development. [3–11]

To realize the health benefits of adequate sleep duration, children are recommended to sleep 10–13 h/night between the ages of 3 and 5 years and 9–11 h/night between the ages of 6 and 13 years [12]. Nevertheless, increasing numbers of children are not meeting these age-specific recommendations [13, 14]. Effective interventions are therefore needed in order to promote adequate sleep duration in children.

The development of effective interventions demands knowing which factors predict sleep duration [15]. As illustrated in Dahlgren and Whitehead’s “determinants of health model,” complex health issues (e.g., sleep) are influenced by factors both within and outside the individual’s control [16]. In this model, individuals with a set of fixed characteristics (i.e., age, sex, hereditary factors) are placed at the center and surrounded by various layers of influences on health. These layers consist of individual lifestyle factors (e.g., physical activity, dietary behavior); social and community networks (i.e., the social context in which the behavior takes place, including family, social and community influences); living and working conditions (e.g., education, poverty, housing); and socioeconomic, cultural, and environmental conditions (e.g., culture and political environment). In addition to their direct effects on health, factors from the various layers have indirect effects as well, as they are interrelated with each other. For example, social factors (e.g., parental rules concerning screen time) are expected to influence sleep health indirectly through such lifestyle factors as screen use before bedtime. Hence, when studying predictors of health, it is important to take all of these layers into account, each within the context of factors from other layers [16]. As such, the Dahlgren and Whitehead model can be used to understand, explain, and predict health behaviors within their real-world contexts. It is therefore applied as the theoretical framework in the current study.

Previous research has revealed that several individual characteristics are related to sleep duration in children. For example, the sleep duration of older children and boys is lower than that of those who are younger or female [17–20]. In addition, individual lifestyle factors—such as the consumption of sugar-sweetened beverages (SSB), screen use and physical activity before bedtime—have been associated with lower sleep duration in children [21–25]. The social and cultural context around children has also been shown to influence sleep duration, with family (parents) playing a particularly important role. Research has indicated that the sleep duration of children with more sleep-related (or other) parental practices was longer than that of other children [26, 27]. Ethnicity might be an important factor as well, given that parental beliefs and attitudes toward the importance of sleep are likely to differ across cultures. For example, previous studies have highlighted differences between Dutch children of migrant and native origin with regard to parenting styles relating to sleep, with children of migrant origin having shorter sleep duration [28–30]. Finally, living conditions, have been found to be associated with sleep duration, especially in the home setting. Previous research has indicated that socioeconomic position (SEP) is associated with short sleep duration in children [18, 31–34]. Much still remains to be understood about how SEP affects sleep, but earlier studies suggest that possible reasons for a disparity in sleep duration are that households with low SEP might have less knowledge about sleep hygiene or other sleep-promoting practices, higher exposure to noise or fewer resources, thus generating a less a healthy sleep environment for children [32–34]. Studies have also suggested that the presence of a television in the bedroom and household size are related to sleep duration. For example, children living in larger households are more likely to share a room with siblings, which has been associated with shorter sleep duration [23, 35–37].

The cross-sectional designs of most of the aforementioned studies [19, 21–23, 25, 26, 28–37] limit the ability to draw conclusion on potential causal relationships between the individual and environmental predictors and sleep duration. In addition, many studies focused on only one or a few potential predictors of sleep duration, thereby ignoring the complexity of sleep health, given that it is influenced by interrelated individual and environmental predictors [18–26, 28–30, 32, 33]. Finally, despite evidence suggesting significant regional differences with regard to sleep duration and the predictors thereof [38], many existing studies were conducted in the USA [22, 23, 26, 27, 31, 35, 36].

Based on these observations, the current study intended to investigate (i) which individual characteristics (i.e., age, sex); individual lifestyle factors (i.e., physical activity behavior, SSB consumption, screen behavior); social and cultural factors (i.e., parental rules for screen use, ethnicity); living conditions (i.e., parental education, presence of a screen in the bedroom, household size); and (ii) which changes in these factors might be associated with the changes in sleep duration seen over time in Dutch primary schoolchildren. In light of existing evidence, we hypothesize that in the univariable analyses, all factors are associated with sleep duration. In the multivariable analyses, however, we predict that the associations of more distal factors (e.g., living conditions and social and cultural factors) become weaker, as they are expected to influence sleep duration through more proximal (e.g., lifestyle) factors.

## Methods

### Study Design and Participants

This study is based on data from the ChecKid study, a dynamic cohort study of primary schoolchildren living in the city of Zwolle, the Netherlands. The study was set up in 2006 and used as a monitor to generate additional insight into trends in the prevalence of overweight and obesity among children aged 4–13 years. Details on the study population and aims of the original ChecKid study have been described elsewhere [39]. ChecKid received ethical approval from the Medical Ethical Committee of the VU University Medical Center Amsterdam (06/243, 2011/411).

Data were collected in three waves: 2006, 2009, and 2012. Every primary school in Zwolle was invited to participate. When a school agreed to be included in the study, all children attending the school, aged 4–13 years, and their parents were invited to participate by a letter distributed by the school. Measurements included a parental questionnaire and anthropometric measurements of the children. In each wave, the children were assessed during the school year in October. In 2009, 34 schools (79%) were willing to participate, with a parental questionnaire and informed consent received from at least one parent for each of 3026 children (35%). In 2012, 35 schools (81%) were willing to participate, with a parental questionnaire and informed consent received from at least one parent for each of 5849 children (61%). Because siblings were allowed to participate in the study, the dataset could contain duplicate family data for different children, thus possibly leading to clustering effects within families. However, most family data (e.g., bedtimes, parenting practices and other variables included) depend on characteristics of the child (e.g., age and lifestyle behaviors of individual children) and not the family as a whole. This is why it was decided to include siblings as well. The current study is based on data from the parental questionnaires from 2009 and 2012. Children who participated both in 2009 and 2012 with complete data for the sleep outcome variable were included (*n* = 1180).

### Measures

The questionnaires included measures of sleep duration, as well as on individual (lifestyle) factors, social and cultural factors, and living conditions. Existing validated questionnaires were used in designing the questionnaire for the ChecKid study, which was adjusted for the current study population. [40, 41]

#### Sleep Duration

Sleep duration was estimated by measuring the sleep window of the child, according to the following questions: “What time does your child usually go to bed on weekdays?” and “What time does your child usually wake up on weekdays?” Parents indicated bedtimes between 6:00 P.M. and 10:30 P.M. and wake-up times between 6:00 A.M. and 8:30 A.M., with precision of 15 min. Information regarding bed- and wake-up times on weekdays was used for the purposes of this study, as it is more constant than bed- and wake-up times on weekends. Hours of sleep were calculated by the hours elapsed between going to bed and waking up. Although these questions did not measure sleep duration directly, parent-reported sleep window is considered an adequate proxy for the estimation of sleep duration [42, 43].

#### Individual Lifestyle Factors

Physical activity behavior was divided into outdoor play on weekdays and involvement in organized sports on weekdays. For both activities, parents could indicate the number of days (i.e., 0–5) and duration per day (i.e., < 0.5, 0.5–1, 1–2, 2–3, or > 3 h/day). Average time spent in outdoor play and organized sports on weekdays was assessed by multiplying the number of days by the mid-category of the indicated duration (i.e., 0.25, 0.75, 1.5, 2.5, 3.5) and dividing the result by five.

Consumption of SSBs—fruit juice, regular soda, sugared tea, and sweetened milk drinks—was assessed according to the number of weekdays (i.e., 0–5) on which the child consumed SSBs and the number of glasses consumed on such days (i.e., < 1, 1, 2, 3, 4, or ≥ 5 glasses/day). Daily SSB consumption was calculated by multiplying the number of days by the number of glasses (i.e., 0.5, 1, 2, 3, 4, 5) and dividing the result by five.

Time spent watching television and using computers/game consoles was assessed according to the number of weekdays on which the child was accustomed to engaging in these activities (i.e., 0–5) and the mean exposure time on such days (i.e., < 0.5, 0.5–1, 1–2, 2–3, or > 3 h/day). Average daily time spent on television and on computers/game consoles was calculated separately by multiplying the number of days by the mid-value of each category (i.e., 0.25, 0.75, 1.5, 2.5, 3.5) and dividing the result by five. Lastly, it was assessed whether the child was accustomed to using a screen (i.e., television/computer/game console) after dinner during a normal school week (yes/no).

#### Social and Cultural Factors

Parenting practices regarding the amount of media use were assessed according to questions asking the parents whether they had rules about (i) how much time the child was allowed to watch television and (ii) how much time the child was allowed to use a computer or game console (yes/no) [26, 27].

Ethnicity was dichotomized as either non-Western background (i.e., one or both parents born in Turkey, Morocco, Suriname, or Netherlands Antilles, as most immigrants in Zwolle are from one of these countries) or Western background (i.e., both parents born in the Netherlands or another unspecified country).

#### Living Conditions

The educational level of the parents was considered, classified as low (lower general secondary education, lower vocational training, and primary school or less), medium (intermediate vocational training, higher general secondary training, and pre-university education), or high (higher vocational training or university), based on the highest level of education completed. For children with two parents, the educational level of both parents was averaged. The parents were also asked if the child had a television, computer, or game console in the bedroom (yes/no). Finally, household size was assessed according to the number of children in the household.

### Change Variables

For all continuous variables (i.e., outdoor play, organized sports, SSB consumption, television use, computer/game console use, household size), change variables were constructed by subtracting the value measured in 2009 from the value measured in 2012. For the dichotomous variables (i.e., presence of a screen in the bedroom, screen use after dinner, parental rules concerning the amount of television, parental rules concerning the amount of computer/game console use), change variables were constructed by recoding the corresponding variables from 2009 and 2012 into a categorical variable with four values, reflecting each of the possible courses (e.g., no screen in the bedroom in 2009 and 2012; adopting a screen in the bedroom between 2009 and 2012; a screen in the bedroom in both 2009 and 2012; a screen in the bedroom only in 2009) [44]. The “Results” section reports only the findings comparing adoption (e.g., of a screen in the bedroom) between 2009 and 2012 to absence (e.g., of a screen in the bedroom) in both 2009 and 2012.

### Statistical Analysis

The data were checked on possible data-entry errors and outliers on the continuous variables. The number of missing values was less than 5% of the total study population and therefore considered inconsequential for the results [45]. Descriptive statistics were calculated to describe the study sample. Differences between the population characteristics in 2009 and 2012 were examined using paired *t* tests for continuous variables and Cochran’s Q tests for dichotomous variables [46]. To identify the associations between the potential predictors and sleep duration, a multivariable linear regression analysis was conducted. All appropriate checks were performed on the assumptions needed to perform the analyses [46]. All relevant assumptions were met.

Two separate models were conducted to investigate the associations between the potential predictors and sleep duration. In both models, sleep duration in 2012 was taken as the dependent variable and all of the analyses were adjusted for sleep duration in 2009. The regression coefficient should therefore be interpreted as the change in sleep duration between 2009 and 2012 [44, 47]. In both models, univariable regression analyses were conducted first, which were adjusted for age, sex, and educational level of the parents, as these factors have been reported to have a strong influence on sleep duration, thus possibly introducing confounding effects [19]. Regression coefficients, 95% confidence intervals (CIs), and *p* values were reported. Any factors showing statistically significant associations with the changes in sleep duration in the univariable analysis were subsequently analyzed using multivariable linear regression, in order to investigate each variable within the context of the others.

In Model 1, the potential predictors measured in 2012 were included as independent variables. Given that a 3-year time interval is relatively long, while most individual lifestyle factors and social and cultural factors have short-term effects on sleep, the predictors measured in 2012 were included in the model, instead of those measured in 2009. This ensured that the association between the predictors in 2012 and changes in sleep duration between 2009 and 2012 was investigated.

In Model 2, the changes in individual (lifestyle), social and cultural factors, living conditions, and changes in sleep duration between 2009 and 2012 were analyzed. These factors were selected, as they are likely to chance within a timeframe of 3 years.

A *p* value of ≤ 0.05 was considered statistically significant. All analyses were executed with IBM SPSS Statistics version 25.

## Results

Data were available for 1180 children, with a mean age of 6.6 ± 1.4 years (range 4–10 years) in 2009 and 9.6 ± 1.4 years (range 7–13 years) in 2012. Mean sleep duration was 11.4 ± 0.5 h/night (range 10–12.75 h/night) in 2009, decreasing by about half an hour to 11.0 ± 0.5 h/night (range 9–12.75 h/night) in 2012. Screen time (i.e., television and computer/game console use, screen use after dinner) and physical activity behavior increased over time, whereas SSB consumption decreased over time. With regard to social and cultural factors and living conditions, 24% of the children had adopted a screen in their bedroom, 17% of the children had adopted rules on the amount of television use, and 20% had adopted rules on the amount of computer/game console use between 2009 and 2012. With the exception of sex, parental education, and ethnicity, all of the variables differed significantly between 2009 and 2012. Further study population characteristics are presented in Table 1.

**Table 1:** Characteristics of the study population in 2009, 2012 and changes in characteristics over this time period

	2009 (*n* = 1180)	2012 (*n* = 1180)	Δ2009–2012
Individual characteristics			
Age in years, mean (SD)	6.6 (1.4)	9.6 (1.4)*	3.0 (0.3)
Sex (female), % (*n*)	52 (602)	^a^	^a^
Daily sleep			
Sleep duration in hours, mean (SD)	11.4 (0.5)	11.0 (0.5)*	− 0.5 (0.5)
Individual lifestyle factors			
Outdoor play in hours, mean (SD)	0.8 (0.6)	0.9 (0.6)**	0.0 (0.6)
Organized sports in hours, mean (SD)	0.2 (0.2)	0.4 (0.3)*	0.2 (0.3)
SSB consumption in glasses, mean (SD)	3.1 (1.6)	2.7 (1.9)*	− 0.4 (1.8)
Television use in hours, mean (SD)	1.0 (0.6)	1.1 (0.7)*	0.1 (0.6)
Computer/game console use in hours, mean (SD)	0.3 (0.3)	0.8 (0.7)*	0.5 (0.6)
Uses a screen after dinner, % (*n*)	58 (688)	87 (1013)*	
Neither in 2009 nor 2012			10 (118)
Adoption between 2009 and 2012			31 (365)
In both 2009 and 2012			56 (648)
Only in 2009			3 (30)
Social and cultural factors			
Ethnicity (non-Western ethnicity), % (*n*)	8 (92)	^a^	^a^
Has rules for the amount of television, % (*n*)	65 (757)	72 (813)*	
Neither in 2009 nor 2012			19 (209)
Adoption between 2009 and 2012			17 (192)
In both 2009 and 2012			55 (618)
Only in 2009			10 (113)
Has rules for the amount of computer/game console, % (*n*)	65 (718)	76 (838)*	
Neither in 2009 and 2012			15 (152)
Adoption between 2009 and 2012			20 (211)
In both 2009 and 2012			56 (578)
Only in 2009			9 (97)
Living conditions			
Parental educational level, % (*n*)			
Low parental educational level	4 (46)	^a^	^a^
Medium parental educational level	28 (321)	^a^	^a^
High parental educational level	68 (770)	^a^	^a^
Presence of a screen in the bedroom, % (*n*)	16 (183)	37 (426)*	
Neither in 2009 and 2012			60 (698)
Adoption between 2009 and 2012			24 (278)
In both 2009 and 2012			12 (144)
Only in 2009			3 (37)
Household size, mean (SD)	2.3 (0.8)	2.4 (0.8)*	0.1 (0.4)

*p < 0.001; **p < 0.05

^a^Considered constant

### Model 1: Associations between individual (lifestyle) factors, social and cultural factors, and living conditions in 2012 and changes in sleep duration between 2009 and 2012

The univariable and multivariable associations between the individual (lifestyle) factors, social and cultural factors, and living conditions measured in 2012 and changes in sleep duration between 2009 and 2012 are presented in Table 2.

**Table 2:** Associations between individual (lifestyle) factors, social and cultural factors and living conditions and changes in sleep duration between 2009 and 2012

	Univariable analysis^*^		Multivariable analysis	
	B (95% CI)	*p* value	B (95% CI)	*p* value
Individual characteristics				
Age	- 0.15 (- 0.17 – - 0.13)	< 0.001	- 0.15 (- 0.17 − - 0.13)	< 0.001
Sex^1^	0.06 (0.01– 0.11)	0.01	0.05 (0.00 – 0.10)	0.04
Individual lifestyle factors				
Outdoor play	0.01 (- 0.03 – 0.05)	0.60	–	–
Organized sports	0.02 (- 0.06 – 0.09)	0.64	–	–
SSB consumption	0.00 (- 0.01 – 0.02)	0.69	–	–
Using a screen after dinner^2^	- 0.11 (- 0.18 – - 0.04)	< 0.01	- 0.10 (- 0.18 − - 0.02)	0.01
Television use	- 0.07 (- 0.10 – - 0.03)	< 0.001	0.00 (- 0.04 – 0.04)	0.99
Computer/game console use	- 0.08 (- 0.11 – - 0.04)	< 0.001	- 0.07 (- 0.11 – - 0.02)	< 0.01
Social and cultural factors				
Ethnicity^3^	0.02 (- 0.07 – 0.10)	0.65	–	–
Rules concerning amount of television use^4^	0.07 (0.02 – 0.12)	0.01	0.04 (- 0.04 – 0.12)	0.33
Rules concerning amount of computer/game console use^4^	0.07 (0.02 – 0.13)	0.01	0.03 (- 0.05 – 0.12)	0.44
Living conditions				
Medium parental educational level^5^	0.01 (- 0.04 – 0.06)	0.62	0.04 (- 0.02 – 0.09)	0.21
Low parental educational level^5^	- 0.21 (- 0.33 – - 0.09)	< 0.001	- 0.15 (- 0.28 – - 0.03)	0.02
Presence of a screen in the bedroom^6^	- 0.04 (- 0.09 – 0.01)	0.14	–	–
Household size	0.03 (0.00 – 0.06)	0.03	0.02 (- 0.01 – 0.05)	0.12

^*^Univariable analyses were adjusted for age, sex and educational level

^1^Reference = Male

^2^Reference = Does not use a screen after dinner

^3^Reference = Western ethnicity

^4^Reference = Has no rules

^5^Reference = High parental educational level

^6^Reference = Does not have a screen in his/her own bedroom

Results of the multivariable regression analysis revealed a significant negative association between age, low parental education (as compared to high parental education), using a screen after dinner, computer/game console use, and changes in sleep duration. For example, more computer/game console use in 2012 was associated with a greater decrease in sleep duration between 2009 and 2012. The significant positive association with sex means that girls had a smaller decrease in sleep duration compared to boys.

### Model 2: Associations between changes in individual (lifestyle) factors, social and cultural factors, and living conditions and changes in sleep duration between 2009 and 2012

The univariable and multivariable associations between changes in individual (lifestyle) factors, social and cultural factors, and living conditions and changes in sleep duration between 2009 and 2012 are presented in Table 3. The multivariable analysis revealed a negative statistically significant association between the changes in computer/game console use and the changes in sleep duration. This means that children with a greater increase in computer/game console use between 2009 and 2012 had a greater decrease in sleep duration in the same time period. No other associations were identified.

**Table 3:** Associations between the changes in individual (lifestyle) factors, social and cultural factors, and living conditions and changes in sleep duration between 2009 and 2012

	Univariable analysis*		Multivariable analysis	
	*B* (95% CI)	*p* value	*B* (95% CI)	*p* value
Individual characteristics				
Age	- 0.15 (- 0.17 – - 0.13)	< 0.001	- 0.15 (- 0.17 – - 0.13)	< 0.001
Sex^1^	0.06 (0.01–0.11)	0.01	0.06 (0.02–0.11)	0.01
Individual lifestyle factors				
Δ Outdoor play	0.00 (- 0.04 – 0.04)	0.84	–	–
Δ Organized sports	0.02 (- 0.06 – 0.09)	0.66	–	–
Δ SSB consumption	0.01 (- 0.01 – 0.02)	0.42	–	–
Δ Using a screen after dinner^2^	- 0.10 (- 0.18 – - 0.01)	0.02	- 0.08 (- 0.16 – 0.01)	0.07
Δ Television use	- 0.02 (- 0.05 – 0.02)	0.41	–	–
Δ Computer/game console use	- 0.08 (- 0.12 – - 0.05)	< 0.001	- 0.08 (- 0.12 – - 0.04)	< 0.001
Social and cultural factors				
Δ Rules concerning amount of television^2^	0.07 (0.00 – 0.15)	0.05	0.07 (0.00 – 0.15)	0.06
Δ Rules concerning amount of computer/game console^2^	0.00 (- 0.07 – 0.07)	0.93	–	–
Living conditions				
Medium parental educational level^3^	0.01 (- 0.04 – 0.06)	0.62	0.03 (- 0.02 – 0.08)	0.29
Low parental educational level^3^	- 0.21 (- 0.33 – - 0.09)	< 0.001	- 0.16 (- 0.28 – - 0.04)	0.01
Δ Presence of a screen in the bedroom^2^	- 0.04 (- 0.08 – 0.01)	0.14	–	–
Δ Household size	0.03 (- 0.03 – 0.08)	0.37	–	–

*Univariable analyses were adjusted for age, sex and parental educational level

^1^Reference = Male

^2^Adoption between 2009 and 2012 compared to neither in 2009 nor 2012

^3^Reference = High parental educational level

## Discussion

The objective of the current study was to investigate (i) which individual characteristics (i.e., age, sex); individual lifestyle factors (i.e., physical activity behavior, SSB consumption, screen behavior); social and cultural factors (i.e., parental rules for screen use, ethnicity); and living conditions (i.e., parental education, screen in the bedroom, household size); and (ii) which changes in these factors might be associated with the changes in sleep duration seen over time in Dutch primary schoolchildren. Mean sleep duration decreased by about half an hour/night between 2009 and 2012. The results further indicate that older children, boys, children who used a screen after dinner, children with increased computer/game console use, and children whose parents had low levels of education had a greater decrease in sleep duration compared to children who were younger, female, did not use a screen after dinner, did not increase their computer/game console use, or whose parents had a high level of education. This article reports on one of the few longitudinal studies that investigated the association between a wide range of predictors and changes in sleep duration.

According to the results, older children had a greater decrease in sleep duration compared to younger children. Although there is a large body of literature about the fact that sleep duration decreases with age, it was not expected that this decrease in sleep duration over time was larger in older children, as this was not reported in previous studies [48–51]. Possible explanations could be that some of these older children start puberty and begin to shift their circadian rhythms or that parents become less involved in their children’s sleep routines and children decide themselves when they actually go to bed [52]. However, more research is needed that could investigate whether there is a (biological) explanation for this finding. Boys had a greater decrease in sleep duration than girls, although the difference was small: the decrease in sleep duration between 2009 and 2012 was 3 min larger for boys compared to girls. This sex difference is in accordance with the results of several previous studies [18–20], although other studies reported no similar association [38, 53]. One possible explanation for sex differences in sleep duration is that parents might be stricter with bedtimes for girls than they are for boys, although evidence for gender-differentiated parenting is mixed [54]. Additional research is needed in order to explore these gender differences, to determine whether they are meaningful and to identify potential explanations, including possible gender-differentiated parenting.

In line with earlier studies [17, 22, 24], the findings of the current study imply that screen use in the evening and increased daily computer/game console use are related to decreased sleep duration. The influence of screen use (particularly in the evening) on sleep duration is generally explained according to three potential underlying mechanisms [23]: pre-sleep arousal, the blue-light process, and displacement. The arousal mechanism suggests that media use in the evening and certain media content (e.g., violent or frightening) causes psychological and physiological arousal, which interferes with the ability to fall and stay asleep, thereby reducing sleep duration [22, 55]. The blue-light process is related to the body’s hormonal response to the light of the screen. Specifically, this mechanism has to do with melatonin—often referred to as the “sleep hormone,” as it increases when darkness falls and subsequently induces sleepiness. Researchers have demonstrated that the shortwave (blue) light emitted from electronic devices (e.g., television screens, mobile phones) can suppress melatonin production, particularly in the evening hours, thus causing a delay in sleep time (i.e., it induces a phase delay in the circadian clock) [56, 57]. Finally, the displacement mechanism refers to the situation in which screen use replaces time that would otherwise have been spent sleeping [58]. This is understood to happen in two ways. First, children postpone going to bed, because they prefer media use to sleep. Second, media use also displaces sleep time (i.e., the time at which the child decides to try to sleep) by being used while in bed [59].

Results of the univariable analyses revealed an association between parental rules concerning media use and changes in sleep duration. After adjusting for other variables in the multivariable analyses, however, this association was no longer significant. One possible explanation is that the association between parental rules and sleep duration is mediated by other factors (e.g., screen use). In other words, children with strict parental rules concerning media use are more likely to have lower levels of screen use and, consequently, be more likely to sleep longer. Several studies have reported an association between parental rules regarding screen time and screen use by children and adolescents [60, 61]. Given that screen use is a relevant predictor of sleep behavior, as also found in the current study, an indirect association might exist. Future research should nevertheless investigate the potential mediating effect of screen use on the association between parental practices and sleep duration.

In the current study, children whose parents had low levels of education had a larger decrease in sleep duration than did those whose parents had higher levels of education. These results are consistent with those of earlier studies, which have identified sleep differences between people with high SEP and those with low SEP [31, 34, 62–64]. Working mechanisms for these health disparities are complicated [65]. For example, children with low SEP might have less favorable sleep hygiene [64, 66] or worse living conditions (e.g., housing, neighborhood) and consequently, be subject to more distractions, noise and stress—all of which can be detrimental to sleep duration [67].

No associations with changes in sleep duration were found for physical activity behavior, SSB consumption, ethnicity, the presence of a screen in the bedroom, or household size. Both SSB consumption and physical activity in the hours before bedtime were reported to have a negative influence on sleep duration [68, 69]. Because the questionnaire used in this study did not specify at what time the child was physically active or consuming such drinks, the lack of association could potentially be explained by timing. The lack of association with ethnic background was not consistent with previous research. This finding might have been due to the fact that only 8% of the children in the study sample were of non-Western background, which is representative of the child population in the city of Zwolle, but not representative of the child population in the Netherlands [39]. The presence or adoption of a screen in the bedroom was not associated with decreased sleep duration in the univariable analysis. This finding was unexpected as the presence of a screen in the bedroom was assumed to be positively associated with screen use in the hours before bedtime and thus with decreased sleep duration [23, 37]. One explanation could be that the questionnaire did not assess whether and at what times children were using the screens in their own bedrooms. Finally, explanations for the lack of association between household size and changes in sleep duration in the univariable analysis could include the narrow variation in household size and the fact that the questionnaire did not assess whether specific children actually did share their bedroom with siblings.

Several strengths and limitations should be taken into account when interpreting the results of this study. The strengths of the study include the large study population and the longitudinal design. Moreover, this research considered a broad range of potential predictors, including environmental factors, whereas other studies focused on either individual characteristics or lifestyle factors (e.g., screen time) [24]. In addressing this broad range of predictors, the current study paints a relatively more complete picture than has been provided by many previous studies and it provides valuable new insights for the development of future interventions. Despite these insights, however, there are still many unexplored factors that could influence short sleep duration in children.

A limitation of the current study is that it was based on parental reports of sleep duration and its potential predictors. Therefore, the results might be affected by recall bias and socially desirable responses, as the parents might have been unaware of the child’s exact behavior. Given the fact that participation was voluntary, non-response response bias could also have occurred. It could be that parents who were interested in their children’s health were more likely to participate in the current study, thus diminishing the potential differences between cultural beliefs, attitudes, and norms. Such selective participation might have led to an underestimation of the results. A second limitation has to do with the measure used for sleep duration. Although the study employed a commonly used and validated questionnaire and similar parental questionnaires are widely used as a measure of sleep duration [18, 30, 48, 49, 70], we did actually measure sleep window wherein sleep onset latency and night wakening were not taken into account. Consequently, by measuring sleep window, actual sleep duration could have been overestimated. Nevertheless, objective measures of sleep duration, such as polysomnography and actigraphy are less usable for studies with large study samples [13, 43]. For these reasons, parent reports of sleep window were deemed to offer an adequate estimation of sleep duration for the current study. As a result, mean sleep duration in the current study was relatively high compared to studies from other countries [18, 47, 48, 53, 71, 72]. At the same time, however, two earlier studies of primary schoolchildren in the Netherlands using a similar measure also identified average sleep duration of more than 10.5 h/night [29] and it has been reported that children from northern Europe sleep longer than children from southern Europe do [38]. Another limitation is the fact that smartphone and tablet use was not assessed. Since the use of such devices have risen drastically in popularity among school-aged children, in particular among the older children, future studies should take this into account and include a more robust assessment of media use. A last limitation of the current study is the fact that only 4% of the study population had low SEP which is not representative of all children in the city of Zwolle and the average population of children in the Netherlands [39]. This distribution might have affected the results of this study, as children with low SEP have been identified as having shorter sleep duration.

Future research should explore whether similar associations related to sleep duration are identified in other contexts, thereby developing a strong base of empirical knowledge for the development of interventions aimed at promoting healthy sleep among primary schoolchildren in the Netherlands. As the sleep duration of all children in the current fell within the recommendations, it should be investigated whether similar results are found in a study population, including children sleeping less than the recommended hours of sleep. In addition, it would be interesting to investigate sleep duration on weekends, as school schedules might limit the variability and influence of the predictors on sleep duration. Another recommendation for future studies is to investigate the underlying working mechanisms of the predictors of lower sleep duration, as identified in this study, including differences in parental education. Given the differences identified for the effects of television viewing and computer use, all future studies should investigate these two factors separately. Finally, given that other dimensions of sleep health (e.g., sleep efficiency and sleep quality) are highly associated with health outcomes, future studies should investigate predictors of these sleep dimensions as well.

If the results of the current study are confirmed in additional studies, future interventions promoting healthy sleep duration in children should be targeted toward priority subgroups, given that relevant differences in sleep duration were found between children whose parents have low levels of education and those whose parents have higher levels of education. In this way, interventions better respect the individual differences of its users and potentially reduces inequalities in sleep health. Secondly, given that sleep health is strongly interrelated with multiple other lifestyle behaviors (e.g., media use) that are influenced by interrelated predictors from the various layers of influences of health, the complex problem of sleep health calls for a system science view. This approach allows for the consideration of relevant social, cultural, and environmental predictors, as well as the potential mediating effects of lifestyle factors, when addressing sleep duration [73].

## Conclusion

This study was one of the first to provide longitudinal sleep data on Dutch primary schoolchildren. The results indicate that mean sleep duration decreased by about half an hour/night between 2009 and 2012. Older children, boys, children who used screens after dinner, children with greater computer/game console use, and children whose parents had low levels of education had a greater decrease in sleep duration.
